# Association between Low Ankle-Brachial Index and Poor Outcomes in Patients with Embolic Stroke of Undetermined Source

**DOI:** 10.3390/jcm11113073

**Published:** 2022-05-29

**Authors:** Minho Han, JoonNyung Heo, Jae Wook Jung, Il Hyung Lee, Joon Ho Kim, Hyungwoo Lee, Young Dae Kim, Hyo Suk Nam

**Affiliations:** 1 Department of Neurology, Yonsei University College of Medicine, Seoul 03722, Korea; umsthol18@yuhs.ac (M.H.); jnheo@yuhs.ac (J.H.); ninja9201@gmail.com (J.W.J.); neu210@yuhs.ac (I.H.L.); joonho345@yuhs.ac (J.H.K.); hwlee25@yuhs.ac (H.L.); neuro05@yuhs.ac (Y.D.K.); 2 Integrative Research Center for Cerebrovascular and Cardiovascular Diseases, Yonsei University College of Medicine, Seoul 03722, Korea

**Keywords:** ankle-brachial index, embolism, prognosis, stroke

## Abstract

We investigated the association of low ankle-brachial index (ABI < 0.9) with major adverse cardiovascular events (MACE) and all-cause mortality in patients with embolic stroke of undetermined source (ESUS) as well as whether the association differed by ESUS subtype. This retrospective single-center study included ESUS patients who underwent transesophageal echocardiography and ABI during hospitalization. ESUS was classified as ESUS with minor cardioembolic source, arteriogenic embolism, two or more causes, or no cause. Arteriogenic embolism was defined and classified as complex aortic or non-stenotic relevant artery plaque. MACE was defined as stroke recurrence, acute coronary syndrome, hospitalization for heart failure, or death. Overall, 829 patients were included, with a median follow-up of 45.8 months. Of these, 42 (5.1%) and 370 (44.6%) had low ABI and arteriogenic embolism, respectively. ABI < 0.9 was independently associated with MACE (hazard ratio [HR]: 2.038, 95% confidence interval [CI]: 1.093–3.801) and all-cause mortality (HR: 3.608, 95% CI: 1.538–8.465) according to the multivariable Cox regression analysis. Between ESUS subtypes, low ABI was independently associated with MACE (HR: 2.513, 95% CI: 1.257–5.023) and all-cause mortality (HR: 5.681, 95% CI: 2.151–15.008) in arteriogenic embolism patients, especially in those with complex aortic plaque. However, in non-arteriogenic embolism patients, low ABI was not related to MACE and mortality. In ESUS patients, low ABI was linked to MACE and all-cause mortality, especially in those with arteriogenic embolisms from complex aortic plaque.

## 1. Introduction

A non-lacunar ischemic stroke without intracranial and extracranial artery stenosis and major-risk cardioembolic sources is known as an embolic stroke of undetermined source (ESUS) [1]. ESUS is accountable for 9–25% of all ischemic stroke cases, and embolism is regarded as the underlying mechanism of these strokes [2]. Two large randomized controlled trials were conducted to investigate the therapeutic effect of anticoagulants targeting hidden embolic sources in patients with ESUS, but those studies did not provide conclusive evidence [3,4]. While a considerable rate of stroke recurrence (~5% in a year) has been reported, reliable prognostic indicators for patients with ESUS have not been established [2]. A reason for this might be the ESUS subtype [5]. ESUS has heterogeneous subtypes, namely, minor cardioembolic source, arteriogenic embolism, two or more causes, and no cause. Further research focusing on ESUS subtypes could expand the current understanding of prognosis in patients with ESUS.

The ankle-brachial index (ABI) is calculated by dividing the ankle systolic pressure by the arm systolic pressure. Low ABI (ABI < 0.9) is a predictor of systemic atherosclerosis and is widely used to diagnose peripheral arterial disease (PAD) [6,7]. Low ABI was also related to increased initial stroke severity and poor outcomes in patients with ischemic stroke [8,9]. Moreover, the prognostic impact of ABI was demonstrated for non-embolic and embolic stroke [10,11]. In this regard, a low ABI may help in identification of high-risk patients with ESUS. However, the prognostic impact of low ABI in ESUS is unknown. Especially, knowledge about the prognostic value of low ABI according to the ESUS subtype has not been studied. Therefore, we aimed to determine if low ABI is linked to prognosis in ESUS. We also investigated whether the association differs among the ESUS subtypes.

## 2. Materials and Methods

### 2.1. Study Population

Patients with acute ischemic stroke who were consecutively registered in the Yonsei Stroke Registry between January 2012 and December 2018 were included. The Cryptogenic Stroke/ESUS International Working Group’s proposed criteria were used to define ESUS [1]. In short, ESUS is diagnosed when findings indicate non-lacunar brain infarction, no proximal artery stenosis, no major-risk cardioembolic source, and no other uncommon cause. Patients with major-risk cardioembolic sources, including atrial fibrillation, atrial flutter, mechanical or bioprosthetic cardiac valve, intracardiac thrombus, recent myocardial infarction (<4 weeks), cardiac tumor, and infective endocarditis or valvular vegetation, were excluded. Patients with uncommon causes of brain ischemia, including cancer-related, reversible cerebral artery vasoconstriction syndrome, and vasculopathy, such as dissection or antiphospholipid syndrome, were also excluded.

ESUS was classified as ESUS with a minor cardioembolic source, with arteriogenic embolism, with two or more causes, or with no cause. Arteriogenic embolism was defined and classified as complex aortic plaque (CAP) or non-stenotic (<50%) relevant artery plaque (NAP) [12,13]. Aortic plaque located in the ascending aorta or the aortic arch with a thickness of ≥4 mm defined CAP [14].

On admission, all patients were examined by brain magnetic resonance imaging and/or computed tomography along with cerebral angiography (magnetic resonance angiography, computed tomography angiography, or digital subtraction angiography). Systemic assessment included 12-lead electrocardiographic monitoring, chest X-ray, standard blood tests, and lipid profiling during stroke unit admission. As a part of the standard evaluation, transesophageal echocardiography (TEE) was performed in all patients, except for those with decreased consciousness, impending brain herniation, poor systemic conditions, inability to accept an esophageal transducer due to swallowing difficulty or tracheal intubation, or lack of informed consent. Moreover, in the included patients, transthoracic echocardiography, heart computed tomography, and Holter monitoring were performed [15]. The stroke subtype was classified according to the Trial of ORG 10172 in the acute classification [16]. In the present study, patients who underwent both TEE and ABI measurements were included (Table S1).

### 2.2. Clinical Variables

Data on baseline characteristics (age, sex, and National Institutes of Health Stroke Scale [NIHSS] score for neurological deficit) upon admission, the presence of risk factors (hypertension, diabetes, hypercholesterolemia, smoking, coronary artery disease, and previous stroke), and laboratory results (total cholesterol, low-density lipoprotein cholesterol, high-density lipoprotein cholesterol, and triglycerides) were collected. Hypertension was defined as systolic blood pressure ≥ 140 mmHg or diastolic blood pressure ≥ 90 mmHg after repeat measurements during hospitalization or current use of antihypertensive medication. Diabetes mellitus was defined as fasting plasma glucose levels ≥ 7.0 mmol/L (126 mg/dL) or taking an oral hypoglycemic agent or insulin. Hypercholesterolemia was defined as serum total cholesterol ≥ 6.2 mmol/L (240 mg/dL), low-density lipoprotein cholesterol ≥ 4.1 mmol/L (160 mg/dL), or any history of use of lipid-lowering agents after a diagnosis of hypercholesterolemia. Current smoking was defined as having smoked a cigarette within 1 year before admission. Coronary artery disease was defined as any cardiac history (acute myocardial infarction, unstable angina, coronary artery bypass graft, or percutaneous coronary artery stent/angioplasty) or the presence of significant stenosis (≥50%) in any of the three main coronary arteries.

### 2.3. ABI Measurement

ABI was measured in the supine position using a previously validated automated device (VP-1000; Colin Co., Ltd., Komaki, Japan) [11]. The device simultaneously measures the blood pressure in the four limbs using the oscillometric method. ABI was calculated as the ratio of ankle systolic blood pressure divided by higher arm systolic blood pressure. After two-sided ABI values were obtained, the lower ABI value was used for the analysis. Low ABI was defined as an ABI < 0.9 [11]. Patients with low ABI were defined as having an ABI < 0.9 on one or bilateral ankles. The mean values of the four-limb blood pressure measurements were also used for analysis.

### 2.4. Follow-Up and Outcomes

Patients included in the study were followed up three and six months after discharge and annually thereafter in the outpatient clinic or through structured telephonic interviews. During follow-up, we assessed functional outcomes, newly developed MACE, newly diagnosed cancer and risk factors, drug compliance, and physical activity. The recurrence of stroke was defined as newly developed neurological symptoms with relevant lesions on brain computed tomography and/or magnetic resonance imaging seven days after index stroke or hospital discharge. When the patients were transferred to another hospital for a major adverse cardiovascular event (MACE), including a recurrent stroke, we attempted to obtain medical records and imaging results. If this was impossible, MACE was determined via telephonic interview. Deaths were confirmed by matching information on death certificates and the identification numbers assigned to patients at birth. In addition, mortality data were obtained based on death certificates from the Korean National Statistical Office (http://www.kostat.go.kr (accessed on 1 January 2020)).

The primary outcome was MACE (defined as any stroke recurrence, acute coronary syndrome, hospitalization for heart failure, or death). The secondary outcome was all-cause mortality. The censoring date was 31 December 2019. If the patient’s last visit was before 31 December 2019, the last visit date was used as the censoring date.

### 2.5. Statistical Analysis

We used SPSS (version 26, IBM, Chicago, IL, USA) for statistical analysis. The statistical significance of intergroup differences was assessed using the χ^2^ test for categorical variables and independent two-sample t-test or the Mann–Whitney U test for continuous variables. Data were expressed as mean ± standard deviation or median (interquartile range) for continuous variables and number (%) for categorical variables. Survival curves were generated according to the Kaplan–Meier method and compared using the log-rank test. Multivariable Cox proportional hazard regression was conducted after adjusting for age, sex, initial stroke severity, risk factors, and variables with *p* < 0.05 in univariable analysis to investigate the independent association between ABI and outcomes. Subgroup analysis was performed using survival curves with log-rank tests; Cox regression analysis was conducted on the basis of the presence and type of arteriogenic embolism. All of the *p* values were two-tailed; *p* values of < 0.05 indicated statistically significant differences.

### 2.6. Standard Protocol Approvals, Registrations and Patient Consent

The Institutional Review Board of Severance Hospital of the Yonsei University Health System approved this study and waived the need for informed consent due to the fact that this study was retrospectively conducted using a prospective database.

### 2.7. Data Availability

The study data are available from the corresponding author upon reasonable request and with permission from all contributing authors.

## 3. Results

### 3.1. Demographic Characteristics

**Figure 1:**
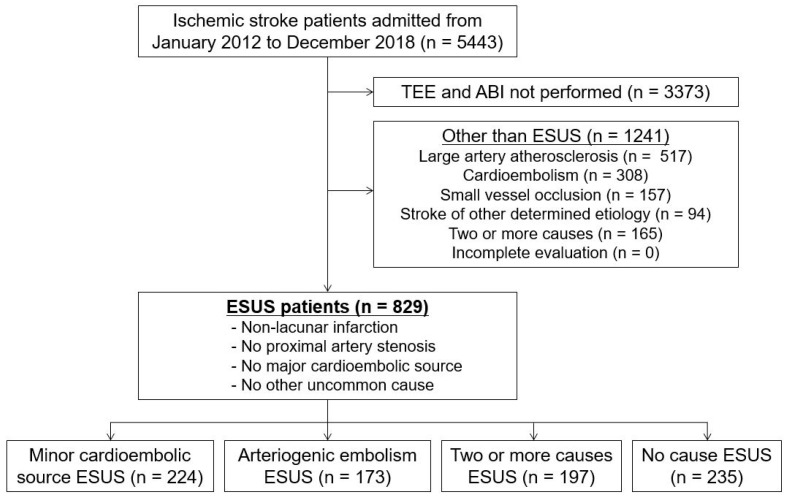
Flowchart of inclusion and exclusion criteria. ABI, ankle-brachial index; ESUS, embolic stroke of undetermined source; TEE, transesophageal echocardiography.

Of the 5443 patients, patients who did not undergo TEE and ABI measurements (*n* = 3373) and those with stroke subtypes other than ESUS, including large artery atherosclerosis (*n* = 517), cardioembolism (*n* = 308), small vessel occlusion (*n* = 157), stroke of other determined cause (*n* = 94), and stroke with two or more causes (*n* = 165) were excluded. Thus, a total of 829 patients with ESUS were included in this study (Figure 1), 224 with minor cardioembolic source (27.0%), 173 with arteriogenic embolism (20.9%), 197 with two or more causes (23.8%), and 235 with no cause (28.3%).

The mean age of patients with ESUS was 63.3 ± 13.1 years; 517 (62.4%) patients were males. The median NIHSS score at admission was 2.0 (interquartile range, 1.0–4.0). Forty-two (5.1%) patients had low ABI; 370 (44.6%) had arteriogenic embolism, including 146 (17.6%) CAP and 224 (27.0%) NAP. Patients with ABI < 0.9 were older, more likely to be male, and more likely to have diabetes and had lower high-density lipoprotein cholesterol, lower brachial diastolic blood pressure, and lower ankle systolic and diastolic blood pressures than those with ABI ≥ 0.9 (all *p* values < 0.05). Arteriogenic embolisms were more frequent in patients with ABI < 0.9 than in those with ABI ≥ 0.9 (71.4% vs. 43.2%, *p* < 0.001). CAP was also more frequent in patients with ABI < 0.9 than in those with ABI ≥ 0.9 (42.9% vs. 16.3%, *p* < 0.001). NAP was identified in 28.6% of patients with ABI < 0.9 and in 26.9% of patients with ABI ≥ 0.9 (*p* = 0.816; Table 1).

### 3.2. Relation of ABI with Long-Term Outcomes

**Figure 2:**
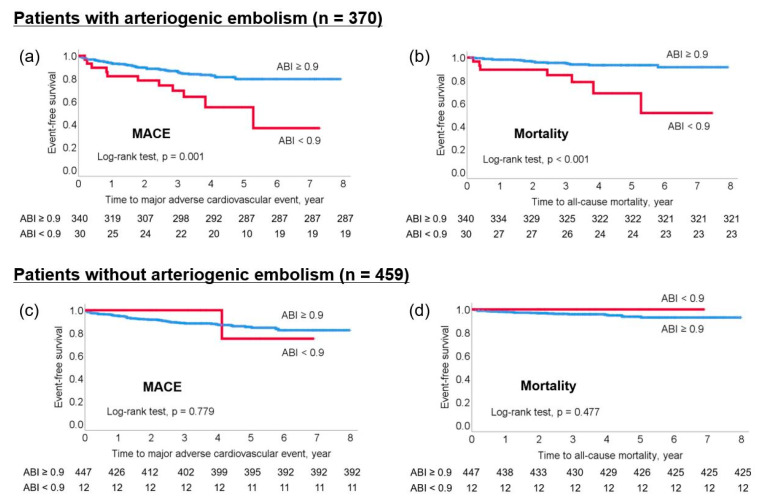
Kaplan–Meier survival analysis. (**a**) MACE and (**b**) all-cause mortality by low ABI in patients with arteriogenic embolism. (**c**) MACE and (**d**) all-cause mortality by low ABI in patients without arteriogenic embolism. ABI, ankle-brachial index; MACE, major adverse cardiovascular event.

Patients were followed up for a median of 45.8 months (interquartile range, 30.2–68.4 months). Overall, 120 patients suffered MACE (14.5%), which included 71 stroke recurrences (8.6%), and 48 (5.8%) died during the study period. The Kaplan–Meier survival curve showed that an ABI < 0.9 was significantly associated both with an increased risk of MACE and all-cause mortality (log-rank test; all *p* < 0.05). Subgroup analysis showed that an ABI < 0.9 was significantly associated with an increased risk of MACE and all-cause mortality in patients with arteriogenic embolism (log-rank test, all *p* < 0.05; Figure 2a,b) but not in those without arteriogenic embolism (Figure 2c,d).

Multivariable Cox proportional hazards regression analysis was performed to adjust for covariates (age, sex, initial stroke severity, hypertension, diabetes, hypercholesterolemia, smoking, coronary artery disease, previous stroke, and high-density lipoprotein cholesterol). A continuous variable of ABI was inversely and independently associated with an increased risk of MACE (hazard ratio [HR]: 0.146, 95% confidence interval [CI]: 0.036–0.592; *p* = 0.007) and all-cause mortality (HR: 0.037, 95% CI: 0.005–0.285; *p* = 0.002). Dichotomized analysis showed that ABI of <0.9 was also independently associated with an increased risk of MACE (HR: 2.038, 95% CI: 1.093–3.801; *p* = 0.025) and all-cause mortality (HR: 3.608, 95% CI: 1.538–8.465; *p* = 0.003). Subgroup analysis for patients with arteriogenic embolism showed that both continuous ABI and ABI < 0.9 were independently associated with an increased risk of MACE (continuous ABI: HR: 0.099, 95% CI: 0.019–0.520, *p* = 0.006; ABI < 0.9: HR: 2.513, 95% CI: 1.257–5.023, *p* = 0.009) and all-cause mortality (continuous ABI: HR: 0.007, 95% CI: 0.001–0.066, *p* < 0.001; ABI < 0.9: HR: 5.681, 95% CI: 2.151–15.008; *p* < 0.001). Conversely, continuous ABI and ABI < 0.9 were associated with neither MACE nor all-cause mortality in patients without arteriogenic embolism (Table 2).

### 3.3. Association between ABI and Outcomes by Arteriogenic Embolism Type

**Figure 3:**
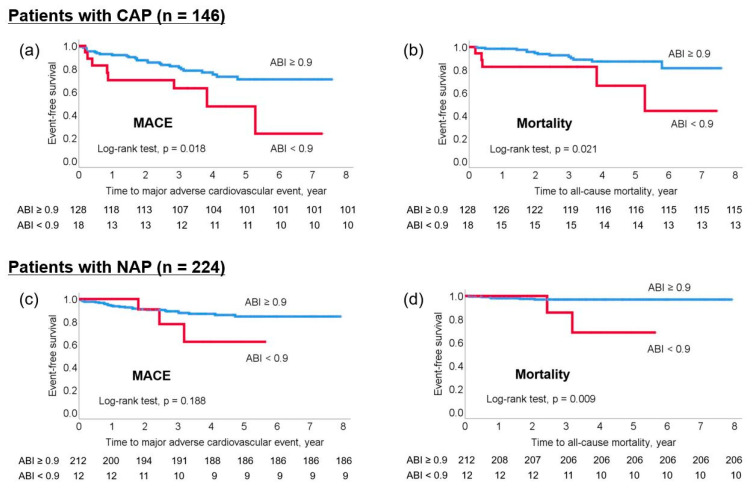
Kaplan–Meier survival analysis. (**a**) MACE and (**b**) all-cause mortality by low ABI in patients with CAP. (**c**) MACE and (**d**) all-cause mortality by low ABI in patients with NAP. ABI, ankle-brachial index; CAP, complex aortic plaque; MACE, major adverse cardiovascular event; NAP, non-stenotic relevant artery plaque.

Three hundred and seventy (44.6%) arteriogenic embolism cases included 146 (17.6%) CAP and 224 (27.0%) NAP cases. The Kaplan–Meier survival curve showed that an ABI < 0.9 was significantly related to an increased risk of both MACE and all-cause mortality in patients with CAP (log-rank test; all *p* < 0.05). In patients with NAP, an ABI < 0.9 was not related to MACE but was associated with all-cause mortality (log-rank test; *p* < 0.05; Figure 3).

In patients with CAP, multivariable Cox proportional hazards regression analysis also showed that continuous ABI and ABI < 0.9 were independently associated with an increased risk of MACE (continuous ABI: HR: 0.043, 95% CI: 0.003–0.587, *p* = 0.018; ABI < 0.9: HR: 2.977, 95% CI: 1.164–7.611, *p* = 0.023) and all-cause mortality (continuous ABI: HR 0.002, 95% CI: 0.000–0.110, *p* = 0.002; ABI < 0.9: HR: 4.812, 95% CI: 1.391–16.647, *p* = 0.013). Whereas, in patients with NAP, continuous ABI and ABI < 0.9 were not associated with MACE but were associated with all-cause mortality (continuous ABI: HR: 0.016, 95% CI: 0.000–0.581, *p* = 0.024; ABI < 0.9: HR: 12.014, 95% CI: 1.613–89.459, *p* = 0.015) (Table 3).

## 4. Discussion

We demonstrated that a low ABI was associated with poor long-term outcomes in patients with ESUS. Low ABI was independently associated with an increased risk of MACE and all-cause mortality particularly in patients with arteriogenic embolism. Moreover, among patients with arteriogenic embolism, only patients with CAP showed an independent association between low ABI and both primary and secondary outcomes. As a result, our research suggests that a low ABI is a useful prognostic marker for patients with ESUS, particularly for those who have CAP-related arteriogenic embolism.

Patients with ESUS are mostly young [17] and have experienced mild stroke [2], as confirmed by our findings. Nevertheless, stroke recurrence rates are reported to be 4.5% per year and mortality rates were 3.9% per year in patients with ESUS [2]. Anticoagulation treatment is a possible alternative to secondary stroke prevention in ESUS, which is characterized by non-lacunar cryptogenic ischemic stroke with embolism as the likely stroke etiology. However, recently, two large randomized clinical trials have refuted this hypothesis [3,4]. A possible reason for these findings may be the heterogeneity of ESUS [18]. Approximately 50% of ESUS cases involved two or more minor-risk embolic sources in an exploratory analysis of the trial [5]. Moreover, studies involving large-scale ESUS cohorts have demonstrated that two-thirds of patients with ESUS had two or more embolic sources [17,19]. Similarly, we found that 44.6% of patients with ESUS had aortic arch or non-stenotic relevant artery atherosclerosis; of these, 23.8% had ESUS with two or more causes. Therefore, the prognosis and predictors of ESUS might vary with the subtype.

According to our study results, a low ABI was independently associated with poor outcomes in ESUS, especially in patients with arteriogenic embolism. Low ABI is a well-established PAD marker [6]. In all subtypes of acute ischemic stroke, a low ABI was independently related to poor short- and long-term outcomes in our prior studies [16,20]. In addition, patients with PAD commonly have concomitant atherosclerosis in the cerebral and coronary arteries due to cardiovascular risk factors that adversely affect all vascular beds [21]. Moreover, we found that patients with low ABI were more likely to be older and have diabetes, lower high-density lipoprotein cholesterol, and arteriogenic embolism. Thus, our study suggests that patients with ESUS and low ABI have a worse prognosis owing to exposure to more vascular risk factors and greater atherosclerotic burden, particularly in the presence of arteriogenic embolism.

Among patients with arteriogenic embolism, only those with CAP showed an association between low ABI and both MACE and mortality. Based on our findings, several hypotheses for the poor prognosis of patients with low ABI and CAP may be suggested. First, CAP is associated with generalized atherosclerosis. We had previously found that CAP was associated with intracranial atherosclerosis [22] and small vessel disease [14]. Other investigators have also reported the association between CAP and coronary artery disease [23]. The accumulated atherosclerotic burden might contribute to a poor prognosis. Second, we found that low ABI and CAP were more frequently observed in older people, and CAP was associated with hypertension and diabetes (Table S2). Unquestionably, age and risk factors are important prognostic markers in patients with acute ischemic stroke [24,25,26]. Third, the annual stroke recurrence rate was reported to be higher in ESUS with CAP than those with NAP (7.2% vs. 5.9%) [27,28]. Moreover, we found that patients with CAP were older, had lower ABI, and had higher frequency of poor long-term outcomes than those with NAP (Table S3). The higher prognostic implication of CAP than that of NAP may explain our results.

Our current study provides strong evidence for the impact of low ABI on ESUS prognosis by enrolling all patients who underwent both TEE and ABI measurements. TEE is the most accurate imaging technique for detecting aortic atherosclerosis and cardioembolic sources with high sensitivity and specificity [29]. The most recent guidelines only suggested that TEE can be useful in the evaluation of ESUS [30]. Due to the clinical importance of CAP in patients with ESUS, our findings suggest that TEE should be more frequently used in these patients to identify potential cardiac or aortic sources of embolism and to estimate ESUS prognosis [31,32].

There are several limitations to this study. First, we excluded a large number of patients due to below reasons. Among the prospective cohort (*n* = 5443), we firstly excluded 3373 (62%) patients who did not undergo both TEE and ABI. The reason why we included patients with both TEE and ABI is described in the previous paragraph. After exclusion, remaining 1241 (22.8%) patients who were not classified as ESUS were excluded according to the definition of ESUS. Second, due to the retrospective design, there may be selection bias. To overcome this, we consecutively included a group of patients with ESUS who underwent TEE. In 93.5% of patients, prolonged heart rhythm evaluations were performed (Table S1). However, variations in the depth of cardiac evaluation and the presence of hidden cardioembolic sources among ESUS subtypes may have affected the results [33]. Third, we measured ABI only once. Repeat measurements are recommended for reliability [34]. To reduce variation in ABI, we repeated tests in patients with ABI < 1.0 or with an inter-arm or ankle blood pressure difference ≥ 10 mmHg. Forth, not all patients underwent angiographic studies for PAD diagnosis. However, ABI is recommended for use in clinical practice and research in accordance with the American College of Cardiology/American Heart Association guidelines [6]. Lastly, this study was conducted using a cohort from a single center in Korea. Further prospective validation studies involving larger cohorts and other ethnicities are needed.

## 5. Conclusions

A low ABI was found to be related to the occurrence of long-term MACE and all-cause mortality in patients with ESUS in this study. The relationship between low ABI and poor long-term outcomes was particularly strong in patients with arteriogenic embolism. Furthermore, only in patients with CAP, a low ABI was linked to MACE and mortality. As a result, ABI measurements could be helpful in predicting the prognosis of ESUS patients, particularly of those with CAP.

## Figures and Tables

**Table 1:** Patient demographic and clinical characteristics.

	Total (*n* = 829)	ABI < 0.9 (*n* = 42)	ABI ≥ 0.9 (*n* = 787)	*p* Value
Age, y	63.3 ± 13.1	67.7 ± 13.0	63.0 ± 13.1	0.024
Men	517 (62.4)	37 (88.1)	480 (61.0)	<0.001
NIHSS score at admission	2.0 [1.0, 4.0]	2.0 [0.0, 4.0]	2.0 [1.0, 4.0]	0.705
Arteriogenic embolism	370 (44.6)	30 (71.4)	340 (43.2)	<0.001
CAP	146 (17.6)	18 (42.9)	128 (16.3)	<0.001
NAP	224 (27.0)	12 (28.6)	212 (26.9)	0.816
Risk factors				
Hypertension	605 (73.0)	31 (73.8)	574 (72.9)	0.901
Diabetes mellitus	237 (28.6)	21 (50.0)	216 (27.4)	0.002
Hypercholesterolemia	151 (18.2)	9 (21.4)	142 (18.0)	0.580
Current smoking	207 (25.0)	15 (35.7)	192 (24.4)	0.099
Coronary artery disease	287 (34.6)	18 (42.9)	269 (34.2)	0.236
Previous TIA/infarction	119 (14.4)	5 (11.9)	114 (14.5)	0.642
Laboratory findings				
Total cholesterol, mmol/L (mg/dL)	4.6 ± 2.1 (179.1 ± 80.5)	4.5 ± 1.3 (173.2 ± 49.4)	4.6 ± 2.1 (179.3 ± 81.7)	0.629
LDL-cholesterol, mmol/L (mg/dL)	2.7 ± 1.0 (105.8 ± 37.4)	2.8 ± 1.1 (109.4 ± 42.4)	2.7 ± 1.0 (105.6 ± 37.2)	0.518
HDL-cholesterol, mmol/L (mg/dL)	1.1 ± 0.3 (43.5 ± 12.8)	1.0 ± 0.3 (38.4 ± 9.8)	1.1 ± 0.3 (43.8 ± 13.0)	0.008
Triglyceride, mmol/L (mg/dL)	1.4 ± 1.1 (126.3 ± 92.6)	1.5 ± 0.8 (131.4 ± 70.3)	1.4 ± 1.1 (125.8 ± 93.6)	0.703
ABI measurements				
Heart rate, bpm	68.7 ± 11.6	67.9 ± 10.8	68.8 ± 11.7	0.626
Brachial SBP, mmHg	145.9 ± 21.8	148.7 ± 20.2	145.8 ± 21.9	0.400
Brachial DBP, mmHg	83.4 ± 13.2	79.4 ± 13.3	83.6 ± 13.2	0.040
Ankle SBP, mmHg	167.0 ± 27.8	135.0 ± 29.7	168.7 ± 26.7	<0.001
Ankle DBP, mmHg	81.9 ± 14.3	70.0 ± 13.8	82.5 ± 14.0	<0.001
ABI	1.09 ± 0.12	0.77 ± 0.12	1.11 ± 0.09	<0.001

ABI, ankle-brachial index; CAP, complex aortic plaque; DBP, diastolic blood pressure; HDL, high-density lipoprotein; LDL, low-density lipoprotein; NAP, non-stenotic relevant artery plaque; NIHSS, National Institutes of Health Stroke Scale; SBP, systolic blood pressure; TIA, transient ischemic attack. Data are expressed as mean ± standard deviation, median [interquartile range], or number (%).

**Table 2:** Multivariable Cox regression analysis of long-term outcomes as per the presence of arteriogenic embolism.

	MACE *		All-Cause Mortality *	
	HR (95% CI)	*p* Value	HR (95% CI)	*p* Value
All patients (*n* = 829)				
ABI	0.146 (0.036–0.592)	0.007	0.037 (0.005–0.285)	0.002
ABI < 0.9	2.038 (1.093–3.801)	0.025	3.608 (1.538–8.465)	0.003
With arteriogenic embolism (*n* = 370)				
ABI	0.099 (0.019–0.520)	0.006	0.007 (0.001–0.066)	<0.001
ABI < 0.9	2.513 (1.257–5.023)	0.009	5.681 (2.151–15.008)	<0.001
Without arteriogenic embolism (*n* = 459)				
ABI	0.423 (0.028–6.376)	0.535	54.379 (0.310–9543.781)	0.130
ABI < 0.9	0.841 (0.111–6.383)	0.867	0.000 (0.000–NA)	0.984

ABI, ankle-brachial index; CI, confidence interval; HR, hazard ratio; MACE, major adverse cardiovascular event; NA, not analyzed. * adjusted for age, sex, National Institutes of Health Stroke Scale score at admission, hypertension, diabetes mellitus, hypercholesterolemia, smoking, coronary artery disease, previous transient ischemic attack/infarction, and high-density lipoprotein.

**Table 3:** Multivariable Cox regression analysis of long-term outcomes according to the characteristics of arteriogenic embolism.

	MACE *		All-Cause Mortality *	
	HR (95% CI)	*p* Value	HR (95% CI)	*p* Value
CAP (*n* = 146)				
ABI	0.043 (0.003–0.587)	0.018	0.002 (0.000–0.110)	0.002
ABI < 0.9	2.977 (1.164–7.611)	0.023	4.812 (1.391–16.647)	0.013
NAP (*n* = 224)				
ABI	0.162 (0.011–2.502)	0.193	0.016 (0.000–0.581)	0.024
ABI < 0.9	2.223 (0.630–7.844)	0.214	12.014 (1.613–89.459)	0.015

ABI, ankle-brachial index; CAP, complex aortic plaque; CI, confidence interval; HR, hazard ratio; MACE, major adverse cardiovascular event; NAP, non-stenotic relevant artery plaque. * adjusted for age, sex, National Institutes of Health Stroke Scale score at admission, hypertension, diabetes mellitus, hypercholesterolemia, smoking, coronary artery disease, previous transient ischemic attack/infarction, and high-density lipoprotein cholesterol.

## Data Availability

Not applicable.

## Supplementary Materials

The following supporting information can be downloaded at: https://www.mdpi.com/article/10.3390/jcm11113073/s1. Table S1: Etiologic evaluations according to ESUS subtypes; Table S2: Patient demographic and clinical characteristics; Table S3: Clinical features of patients with arteriogenic embolism.
