# Supplementary material for: Association between Low Ankle-Brachial Index and Poor Outcomes in Patients with Embolic Stroke of Undetermined Source
Source: J Clin Med. 2022 May 29;11(11):3073. doi: 10.3390/jcm11113073 (PMC9181204; doi:10.3390/jcm11113073)
Supplement: Supplementary file 1 [file jcm-11-03073-s001.zip › jcm-1710956-supplementary.pdf]

**Table S1. Etiologic evaluations according to ESUS subtypes.**

| | Total (n = 829) | Arteriogenic embolism (n = 173) | Minor cardioembolic source (n = 224) | No cause (n = 235) | Two or more causes (n = 197) | *p* value |
|---|---|---|---|---|---|---|
| Angiographic evaluations | 798 (96.3) | 166 (96.0) | 221 (98.7) | 216 (91.9) | 195 (99.0) | <0.001 |
| CTA | 594 (71.7) | 123 (71.1) | 169 (75.4) | 153 (65.1) | 149 (75.6) | 0.044 |
| MRA | 741 (89.4) | 150 (86.7) | 210 (93.8) | 199 (84.7) | 182 (92.4) | 0.004 |
| DSA | 109 (13.1) | 26 (15.0) | 22 (9.8) | 28 (11.9) | 33 (16.8) | 0.154 |
| Neurosonographic evaluations | 795 (95.9) | 164 (94.8) | 219 (97.8) | 217 (92.3) | 195 (99.0) | 0.002 |
| Carotid Doppler | 735 (88.7) | 154 (89.0) | 195 (87.1) | 205 (87.2) | 181 (91.9) | 0.376 |
| TCD | 747 (90.1) | 150 (86.7) | 210 (93.8) | 206 (87.7) | 181 (91.9) | 0.049 |
| Echocardiography | | | | | | |
| TEE | 829 (100) | 173 (100) | 224 (100) | 235 (100) | 197 (100) | NA |
| TTE | 742 (89.5) | 155 (89.6) | 210 (93.8) | 196 (83.4) | 181 (91.9) | 0.002 |

| | | | | | | |
|---|---|---|---|---|---|---|
| Heart rhythm evaluations | 775 (93.5) | 161 (93.1) | 205 (91.5) | 225 (95.7) | 184 (93.4) | 0.328 |
| Continuous ECG monitoring | 709 (85.8) | 148 (85.5) | 185 (82.6) | 205 (87.2) | 171 (86.8) | 0.499 |
| Holter | 419 (50.5) | 85 (49.1) | 113 (50.4) | 139 (59.1) | 82 (41.6) | 0.004 |
| Implantable loop recorder | 9 (1.1) | 2 (1.2) | 1 (0.4) | 6 (2.6) | 0 (0.0) | 0.052 |
| Heart CT | 545 (65.7) | 103 (59.5) | 173 (77.2) | 142 (60.4) | 127 (64.5) | <0.001 |
| ABI | 829 (100) | 173 (100) | 224 (100) | 235 (100) | 197 (100) | NA |

Abbreviation: ABI, ankle-brachial index; CT, computed tomography; CTA, computed tomography angiography; DSA, digital subtraction angiography; ECG, electrocardiogram; NA, not analyzed; TCD, transcranial Doppler; TEE, transesophageal echocardiography; TTE, transthoracic echocardiography. Data are expressed as number (%).

**Table S2. Patient demographic and clinical characteristics.**

| | CAP (n = 146) | Non-CAP (n = 683) | *p* value |
|---|---|---|---|
| Age, y | 67.0±10.9 | 61.8±13.1 | <0.001 |
| Men | 107 (73.3) | 410 (60.0) | 0.003 |
| NIHSS score at admission | 2.0 [0.0, 4.0] | 2.0 [1.0, 4.0] | 0.538 |
| **Risk factors** | | | |
| Hypertension | 122 (83.6) | 483 (70.7) | 0.002 |
| Diabetes mellitus | 52 (35.6) | 185 (27.1) | 0.038 |
| Hypercholesterolemia | 29 (19.9) | 122 (17.9) | 0.570 |
| Current smoking | 31 (21.2) | 176 (25.8) | 0.250 |
| Coronary artery disease | 56 (38.4) | 231 (33.8) | 0.296 |
| Previous TIA/infarction | 22 (15.1) | 97 (14.2) | 0.786 |
| **Laboratory findings** | | | |
| Total cholesterol, mg/dL | 172.0±47.6 | 180.5±85.8 | 0.244 |
| LDL-cholesterol, mg/dL | 103.9±38.0 | 106.2±37.3 | 0.501 |
| HDL-cholesterol, mg/dL | 42.1±10.6 | 43.9±13.3 | 0.116 |
| Triglyceride, mg/dL | 134.2±119.0 | 124.3±85.8 | 0.236 |

Abbreviation: CAP, complex aortic plaque; HDL, high-density lipoprotein; LDL, low-density lipoprotein; NIHSS, National Institutes of Health Stroke Scale; TIA, transient ischemic attack.

Data are expressed as mean ± standard deviation, median [interquartile range], or number (%).

**Table S3. Clinical features of patients with arteriogenic embolism.**

| | CAP (n = 146) | NAP (n = 224) | *p* value |
|---|---|---|---|
| Age, y | 67.0±10.9 | 64.6±11.7 | <0.001 |
| Hypertension | 122 (83.6) | 176 (78.6) | 0.236 |
| Diabetes mellitus | 52 (35.6) | 70 (31.3) | 0.383 |
| Hypercholesterolemia | 29 (19.9) | 46 (20.5) | 0.875 |
| Current smoking | 31 (21.2) | 62 (27.7) | 0.162 |
| Coronary artery disease | 56 (38.4) | 98 (43.8) | 0.304 |
| Previous TIA/infarction | 22 (15.1) | 40 (17.9) | 0.483 |
| ABI | 1.06±0.15 | 1.09±0.12 | 0.020 |
| ABI < 0.9 | 18 (12.3) | 12 (5.4) | 0.016 |
| All-cause mortality | 18 (12.3) | 8 (3.6) | 0.001 |
| MACE | 35 (24.0) | 29 (12.9) | 0.006 |

Abbreviation: ABI, ankle-brachial index; CAP, complex aortic plaque; NAP, non-stenotic relevant artery plaque; MACE, major adverse cardiovascular event; TIA, transient ischemic attack. Data are expressed as mean ± standard deviation or number (%).
